# Differences Between the Strength of Preference–Performance Coupling in Two Rice Stemborers (Lepidoptera: Pyralidae, Crambidae) Promotes Coexistence at Field-Plot Scales

**DOI:** 10.1093/ee/nvab034

**Published:** 2021-04-28

**Authors:** Finbarr G Horgan, Angelita M Romena, Carmencita C Bernal, Maria Liberty P Almazan, Angelee Fame Ramal

**Affiliations:** 1 EcoLaVerna Integral Restoration Ecology, Bridestown, Kildinan, Co. Cork, Ireland; 2 Universidad Católica del Maule, Facultad de Ciencias Agrarias y Forestales, Escuela de Agronomía, Casilla 7-D, Curicó, Chile; 3 Environment and Sustainable Resource Management, University College Dublin, Belfield, Dublin 4, Ireland; 4 International Rice Research Institute, DAPO Box 7777, Metro Manila, Philippines; 5 School of Environmental Science and Management, University of the Philippines, Los Baños, 4030 Laguna, Philippines

**Keywords:** *Chilo*, host preference, integrated pest management, Philippines, rice resistance, *Scirpophaga*

## Abstract

Two stem-boring moths, the yellow stemborer (YSB) *Scirpophaga incertulas* (Walker), and the striped stemborer (SSB), *Chilo suppressalis* (Walker), damage rice in Asia. YSB is the dominant species in much of tropical Asia. Both species are oligophagous on domesticated and wild rice. We investigated the roles of host plant preferences and larval performance in determining the larval densities of both species in rice plots. In screenhouse experiments, YSB showed significant preference–performance coupling. Adults preferred high-tillering rice varieties during early vegetative growth. In contrast, SSB did not demonstrate oviposition preferences under the same screenhouse conditions, but did oviposit less on the wild rice *Oryza rufipogon* Griff. than on domesticated rice varieties during a choice experiment. Despite differences in preference–performance coupling, larval survival and biomass across 10 varieties were correlated between the two species. YSB and SSB larvae occurred in relatively high numbers on rice varieties with large tillers (IR70, IR68, and T16) in wet and dry season field experiments. However, whereas YSB was the dominant species on IR68 and IR70, it was relatively less abundant on T16, where SSB dominated. Results suggest that YSB preferentially attacked fast-growing rice varieties with high tiller numbers early in the crop cycle. Meanwhile SSB, which has weak preference–performance coupling, occurred in rice plants with large tillers that were relatively free of YSB later in the crop cycle. These factors may allow the species to coexist. We discuss the implications of proximate and ultimate factors influencing stemborer co-occurrence for the sustainable production of rice in tropical Asia.

Rice is attacked by a range of herbivorous insects that cause losses in paddy profitability from farm to regional scales. Among the principal pests of rice in Asia are a number of lepidopteran stemborers: these include the yellow stemborer (*Scirpophaga incertulas* (Walker) [YSB]), the striped stemborer (*Chilo suppressalis* (Walker) [SSB]), the pink stemborer (*Sesamia inferens* (Walker) [PSB]), and the white stemborer (*Scirpophaga innotata* (Walker) [WSB]) (Heinrichs 1994, Litsinger et al. 2006). These stemborers will often co-occur in rice as complexes of two or more species over their distribution ranges. Furthermore, different species will dominate stemborer assemblages depending on the region. For example, WSB is the predominant species attacking rice in Mindanao (Philippines) and Java (Indonesia) (Litsinger et al. 2006), YSB is the predominant species in Luzon (Philippines) (Zhu et al. 2002, Horgan et al. 2020), and SSB the predominant species in north east Asia (Kiritani 1990).

Large-scale changes in rice production practices can cause shifts in the compositions of regional stemborer assemblages, including changes in species dominance. For example, SSB replaced YSB as the dominant species in southern Japan during the 1950s (Kiritani 1990), and WSB replaced YSB as the dominant species in Mindanao and Java in the 1980s (Litsinger et al. 2006). These shifts in dominance have been partly attributed to changes in rice production practices, including improved irrigation, changes from single to double cropping, changes from staggered to synchronized planting, and the wide-scale adoption of improved rice varieties (Kiritani 1990, Litsinger et al. 2006, Horgan and Crisol 2013, Horgan 2020). Stemborer oviposition preferences and their comparative performance (survival, growth, and fecundity) on different rice hosts (varieties, growth stages, nutrient contents) may further explain stemborer distributions across rice fields, and could help predict potential shifts in species dominance following regional changes in crop production practices.

A number of studies have indicated that adult rice stemborers will select the most suitable rice varieties for larval development (e.g., *Maliarpha separatella* Ragonot: (Malinga 1985); *Diatraea saccharalis* (Fabricius): (Sidhu et al. 2013); SSB: (Shim 1965)). However, there have been discrepancies as to the strength and nature of preference–performance relations across studies, even for the same stemborer species. For example, Shim (1965) indicated marked preference–performance coupling for SSB, but Jiang et al. (2015) failed to detect any significant preference–performance coupling in their study with the same moth species. Several studies have failed to detect any relation between preference and performance of YSB in controlled experiments (Chandramohan 1983, Riaz et al. 1993, Zhu et al. 2002, Horgan et al. 2020).

Strong preference–performance coupling will ensure that a species finds the most suitable hosts for its offspring. However, strong preferences may also have associated trade-offs where several species occupy similar niches. For example, because stemborer species are expected to select their preferred hosts based on the same host anatomical and nutritional requirements for developing larvae, then strong, shared preferences among coexisting species may be expected to promote interspecific aggregation and consequent competition. In contrast, differences in host preferences, or in the relative strengths of preference–performance coupling between species might promote coexistence. As a corollary, strong preference–performance coupling might be associated with a greater competitive ability, and weak coupling with a greater ability to disperse or otherwise avoid competition (Augustyn et al. [2016], but see Gripenberg et al. [2010]). Such differences in the relative strengths of preference–performance coupling might also create a disconnect between proximate host preferences and ultimate larval distributions at field (1000s of m^2^) or plot scales (100s of m^2^) under the influence of interspecific stemborer interactions.

In this study, we compared preference–performance relations in YSB and SSB across 12 rice lines in screenhouse and field experiments. Both stemborers are oligophagous species although relatively monophagous strains (populations) of each species may occur (Arvind 1987, Cohen and Cuong 2002, Jiang et al. 2015, Cuong et al. 2016). Both species deposit egg masses from which tens of neonates disperse to attack rice tillers. The larvae develop inside the rice tillers, apparently with little post-establishment dispersal (Zhu et al. 2002, Horgan et al. 2020). To improve on previous study designs that investigated the preferences of ovipositing moths on plants grown under one set of conditions and the performance of stemborer larvae using plants grown under a different set of conditions (Riaz et al. 1993, Zhu et al. 2002, Horgan et al. 2020), we conducted our oviposition choice tests in microplots and examined larval performance in the same microplots after the eggs had hatched (i.e., preference and performance were assessed on the same individual plants without breaking the insect life cycle).

In one set of experiments, we used 10 rice varieties with known variability in resistance to stemborers. In a second set of experiments, we used two varieties together with a relatively resistant rice species, *Oryza rufipogon* Griff.; we included *O. rufipogon* because of a growing interest in developing resistant rice varieties through interspecific hybridization between domesticated rice and this species (Reuolin et al. 2019, Sarangi and Islam 2019). We also compared egg laying and larval distribution across field plots to assess the utility of experiments conducted at plant or microplot scales in determining field damage from complexes of stemborer species. We predicted that the species displaying stronger preferences would occur on the most suitable host varieties in the field, with the second species occupying relatively competitor free-plants. To our knowledge, this is the first study to compare preference–performance relations in YSB and SSB across the same rice varieties.

## Methods

### Study Species

We used YSB and SSB in our experiments. In field experiments, we also observed PSB, but at relatively low numbers. For experiments under controlled conditions, we collected adult YSB from farmers’ fields in Laguna, Philippines. Adult SSB were collected from the International Rice Research Institute (IRRI) Experimental Field Station at Los Baños, Philippines. Moths were collected using entomological nets during late evenings on the day prior to use in oviposition experiments. To acquire larvae for experiments, the egg masses were collected from the oviposition experiments (see below), measured using calipers, and maintained in individual glass vials until neonates emerged. The numbers of neonates emerging from each egg mass were recorded and the masses examined for unhatched eggs. Neonates were used in experiments within 1 h of egg hatch.

### Plant Materials

We used 10 rice varieties in our main experiments. The rice varieties TKM6, Taitung 16 (T16), IR36, IR40, IR50, IR62, IR66, IR68, IR70, and IR72 were selected based on unpublished records from IRRI of varietal screening for resistance development to stemborers (IRRI, unpublished). The screening tests indicated a range of susceptibilities to stemborers among the varieties. In a second group of experiments, we used the varieties IR62 and IR64, and the wild rice species *O. rufipogon*. Because of restrictions on experiments with wild rice species in open fields in the Philippines, we only conducted experiments with the second group of rice lines under controlled, screenhouse conditions. Seed was acquired through the IRRI Germplasm Bank with IR varieties acquired through the Plant Breeding, Genetics and Biotechnology Division of IRRI. For screenhouse studies, the seed was initially sown to seed boxes with fine soil and transplanted to paddy soil after 9 d. For field experiments, the seed was sown to raised seedbeds in a screenhouse and transplanted to field plots after 28 d.

### Stemborer Preferences for Rice Varieties

Oviposition choice experiments were conducted in a screenhouse facility. The screenhouse consisted of concrete-walled, mud-filled trenches that simulate field conditions, but without much of the insect and disease pressures of field conditions because the house was clad with 1 mm insect netting. We conducted three separate oviposition experiments. In the first two experiments, SSB (experiment 1) or YSB (experiment 2) females were allowed to freely oviposit on 10 rice varieties (IR36, IR40, IR50, IR62, IR66, IR68, IR70, IR72, T16, and TKM6). In experiment 3, females of SSB and YSB (in separate cages) were allowed to freely oviposit on three rice lines (IR62, IR64, and *O. rufipogon*). The experiments were conducted as follows.

Seedlings (15 d old) of each variety were transplanted at one seedling per hill to microplots in the screenhouse facility. In experiments 1 and 2, each microplot consisted of 30 hills (10 varieties × 3 hills) distributed as 5 rows with a spacing of 35 × 35 cm between hills. The varieties were randomized within each microplot. The microplots were individually covered with mesh cages of dimensions 2.45 × 2.10 × 1.4 m (L × W × H) replicated six times. In experiment 3, microplots consisted of one plant of each of the three test lines planted side-by-side with 25 cm spacing. Plants were randomized among the three positions and the plots were covered with mesh cages of dimensions 1.0 × 0.5 × 1.4 m (L × W × H). The entire experiment consisted of 12 cages (2 moth species × 6 replicates).

Fertilizer was applied to plots in all experiments at a rate of 100 kg of nitrogen/hectare 10 d after transplanting (DAT). At 20 DAT, the cages were each infested with 25 (experiments 1 and 2) or 2 (experiment 3) gravid females of either YSB or SSB. After 5 d, the cages and adults were removed and the plants were searched for egg masses. The number of egg masses per hill was noted and the masses collected and individually stored in scintillation vials to allow larvae to emerge. The numbers of larvae emerging from each egg mass were recorded.

### Stemborer Performance on Rice Varieties

We assessed stemborer performance in the same microplots that were used in the choice oviposition experiments (see above). The protocol for each of the three experiments was the same. At 35 DAT (mid-tillering), when all egg masses had been removed from the microplots, each plant was infested with 10 neonate YSB or SSB using a fine paintbrush. Neonates were carefully placed on individual tillers (one per tiller), above the water line. The delay in infesting, after removal of all egg masses, was determined by the time for neonates to emerge from the egg masses (as observed in the laboratory). After infestation, the larvae were allowed to develop on the plants for 35 d after which the plants were destructively sampled. Plants were pulled from the soil and the numbers of tillers, including dead tillers (dead heart), were recorded. The stems were carefully examined for larval entry holes and were dissected to collect developing larvae and pupae. The larvae and pupae were counted, dried, and weighed. Survival was calculated as the proportion of the original 10 neonates per plant that were found alive. Development was measured as the proportion of larvae than had developed to the pupal stage at the time of sampling. The plants were placed in paper bags and dried in a forced draught oven at 60°C for 2 wk before being weighed.

### Preference and Performance in Field Experiments

We conducted two field experiments with natural stemborer infestations to examine preferences and performance of stemborers in field plots with different test varieties. In both of the field trials, we focused on early crop stages (≥60 DAT – seedling to booting). The experiments were conducted during the wet and dry season at the Experimental Field Station of IRRI. The field site at IRRI had deep clay soils with about 4% organic matter. The experiments each consisted of field plots of <104 m^2^ with different rice varieties. Plots were flooded until pre-harvest and received fertilizer (ammonium sulphate) equivalent to 100 kg (wet season) or 150 kg (dry season) of nitrogen per hectare as a basal treatment, at 3 wk after transplanting, and again at panicle initiation. Solophos, muriate of potash and zinc were applied basally with the ammonium sulphate. Plots received no pesticide treatments. Seed was initially sown to screen house seedbeds and 28-d old seedlings transplanted as one plant per hill to the puddled field plots. Hills were spaced at 25 × 25 cm (planting distance). Further details of each experiment are presented below.

### Field Screening of 10 Varieties During the Wet Season

During the wet season, field plots of 5 × 5 m were planted with seedlings of one of each of the 10 rice varieties that were used in oviposition choice experiments 1 and 2 described above (i.e., IR36, IR40, IR50, IR62, IR66, IR68, IR70, IR72, T16, and TKM6). The plots were planted as six replicated blocks (completely randomized block design) with five rice hills of TN1 between adjacent blocks.

The rice plots were examined at 15 DAT (early tillering) for stemborer egg masses by carefully examining each hill. Rice plots were randomly sampled for pest and disease incidence at 30 DAT (mid-tillering) and 60 DAT (booting) (see Supp Information [online only] for details on other pests and diseases). At each sampling point, a single rice hill was carefully pulled and stored in a plastic pouch. At the same time that plants were randomly sampled, a second, undamaged plant was collected from each field plot. Undamaged plants were selected after field examination of tillers for stemborers failed to indicate evidence of attack.

All plants were examined in the laboratory noting the number of vegetative and reproductive tillers and dissecting each tiller to search for stemborer larvae. Leaves were examined for insect damage and diseases, noting the numbers of leaves affected and the total number of leaves per plant. Stemborers were collected from the sampled plants and were identified (species and life stage) and weighed (total biomass per plant). After examination in the laboratory, the plants were separated into different parts (roots, shoots, and panicles) and dried at 60°C in a forced draught oven until a stable weight.

### Field Screening of Four Varieties During the Dry Season

During the dry season field experiment, 5 blocks, each with 4 plots of 8.25 × 12.5 m (L×W) were planted with IR66, IR68, IR70, and IR74 (one variety per plot, 20 plots = 4 varieties × 5 replicated blocks) in a randomized block design. The varieties were selected on the basis of relatively low (IR66 and IR72) and high (IR70 and IR68) stemborer damage (i.e., whitehead panicles) in the previous wet season field experiment. The plots were examined at 15 DAT (early tillering) for stemborer egg masses. Individual plants were randomly sampled from each plot for stemborer incidence at 30 DAT (mid-tillering) and 60 DAT (booting), and the plants were processed in the laboratory as described above. No control, stemborer-free rice plants were selected from plots during the dry season experiment.

### Data Analyses

The relations between egg mass length and the numbers of eggs per mass were analyzed using linear regression. Results from screenhouse choice oviposition experiments (egg mass size, egg masses, and emerging larvae) were analyzed separately for each stemborer species using univariate general linear models (GLM) with variety as the main factor and removing the effects of blocks. Data were ranked because of nonindependence of measures. Stemborer performance on the rice lines in the screenhouse was analyzed using GLM with one (variety) factor for damage, survival, total insect biomass and development times or two factors (variety, sex) for pupal weights, and removing the effects of blocks. Tiller number was initially included in the analysis as a covariate, but was removed where there was no significant effect. We conducted a range of Spearman’s correlations between the results from the screenhouse oviposition and larval performance experiments to assess preference–performance relations for each stemborer species. Spearman’s correlations were also used to examine relations between oviposition and performance of the two stemborer species and to assess the relations between tiller number and plant weight on stemborer fitness (preference and performance). Results (stemborer numbers) from the field trials were analyzed using multivariate analysis of variance (MANOVA) that included YSB, SSB, and PSB larval numbers as dependent variables. Variety was the main factor examined and block effects were removed. MANOVAs were conducted separately for each sampling date. Tukey’s HSD tests were conducted for all significant GLM and MANOVA results. Following analyses, residuals from each parametric test were plotted and assessed for normality and homogeneity. Data transformations are indicated with statistical results. Multiple regression with backward elimination was used to determine the best predictors of field infestations during the wet season experiment. We used data from the noninfested plants (tiller number, stem weight, tiller weight, plant height, total leaf weight, the number of leaves per tiller, and panicle weight) as independent variables and larval numbers as dependent variables. We also included the combined numbers of heterospecific stemborer larvae, whorl maggot damage, and damage from other lepidopterans as independent variables at the initiation of each analysis. Residuals were plotted following the analyses to ensure normality and homogeneity of variances.

## Results

### Egg Densities in Masses

**Fig. 1.**
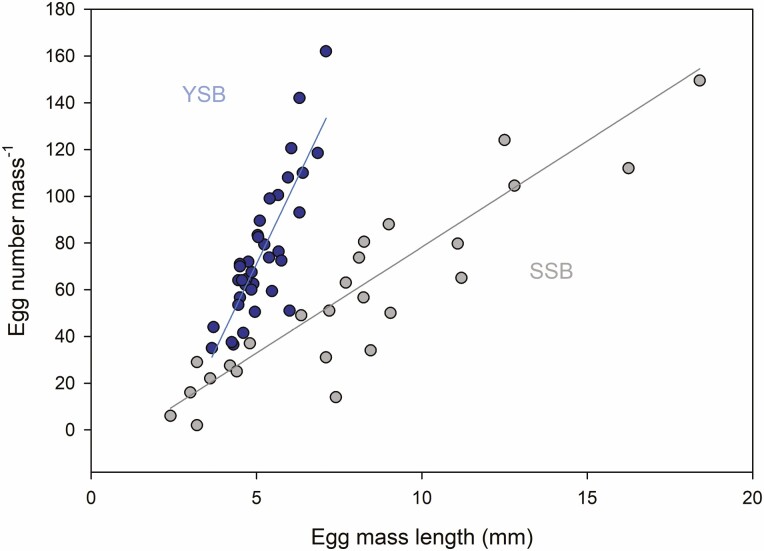
Relation between the lengths of egg masses and the number of eggs in each mass for YSB (blue symbols) and SSB (gray symbols). Linear models are indicated by blue (YSB: y = 29.500x-76.827) and gray (SSB: y = 9.0726x-12.44) lines. See online version for colors.

YSB egg masses were shorter than the egg masses of SSB; however, the YSB egg masses generally contained more eggs (*F*_1,60_ = 48.416, *P* < 0.001; covariate length *F*_1,60_ = 95.959, *P* < 0.001). Egg mass length was related to egg number in both species with a greater slope for YSB (YSB: y = 29.50x-76.83; *R*^*2*^ = 0.69; *P* < 0.001; SSB: y = 9.07x-12.44; *R*^*2*^ = 0.76; *P* < 0.001; Fig. 1).

### Stemborer Oviposition Preferences

**Fig. 2.**
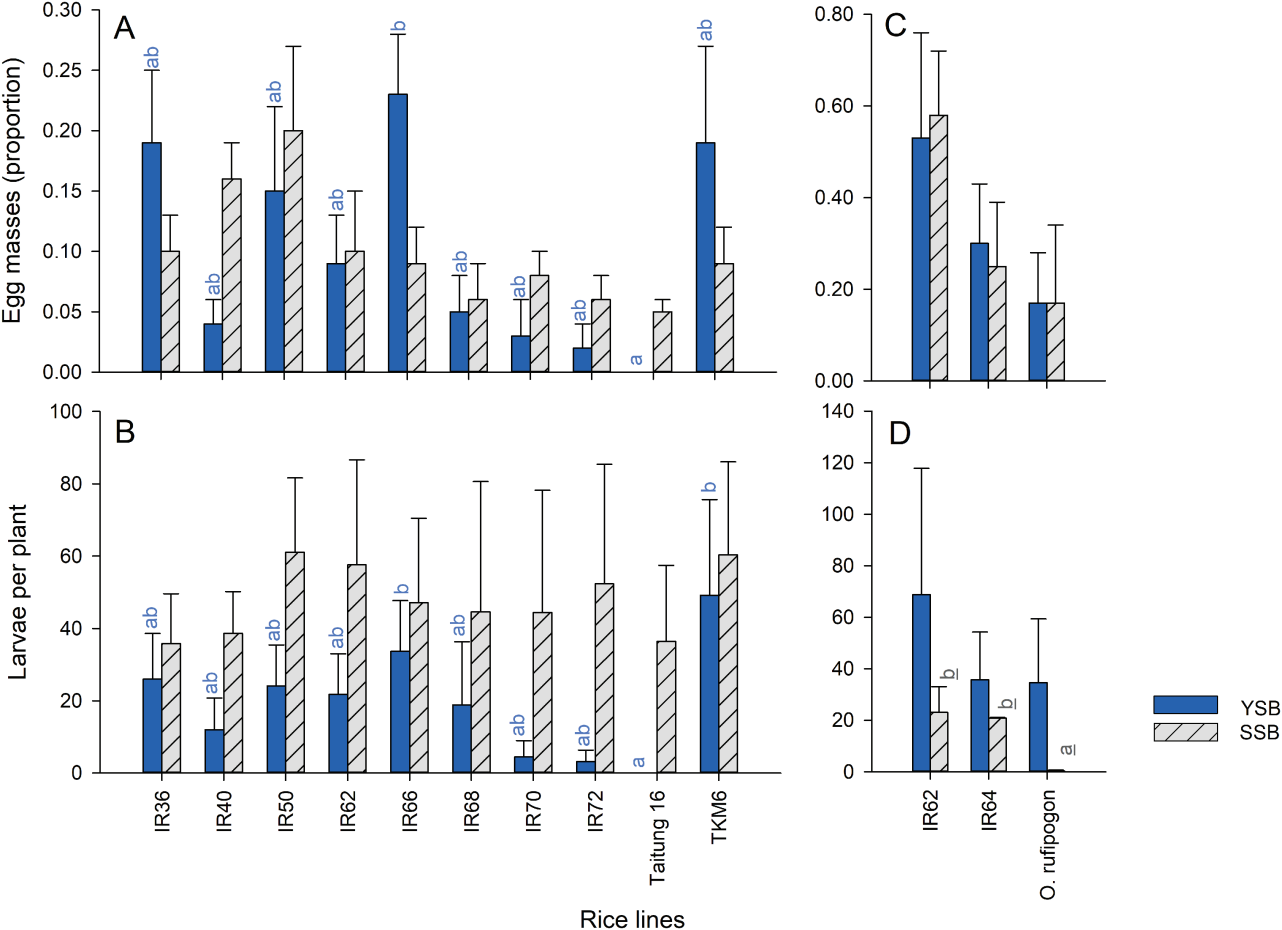
Results from choice oviposition experiments with YSB (blue bars) and SSB (hatched, gray bars). The average numbers of egg masses deposited (A) and the number of larvae emerging from the masses (B) are indicated for 10 rice varieties. The average numbers of egg masses deposited (C) and the number of larvae emerging (D) from an experiment with three rice lines are also shown. Standard errors are indicated, lowercase letters indicate homogenous groups based on analyses of ranked data (blue = YSB, gray, underlined = SSB: Tukey, *P* > 0.05; *n* = 6 for each species × variety group). See online version for colors.

YSB and SSB differed in their preferences for rice varieties in the choice oviposition experiments. Whereas YSB demonstrated a preference for IR66 plants and avoided T16 plants (*F*_9,50_ = 3.256, *P* < 0.01: Fig. 2A), SSB showed no significant preferences across varieties (*F*_9,50_ = 0.897, *P* > 0.05: Fig. 2A). Similar results were noted when the numbers of larvae that emerged from the egg masses were analyzed (YSB: *F*_9,50_ = 2.893, *P* < 0.05; SSB: *F*_9,50_ = 0.576, *P* > 0.05: Fig. 2B).

We found no significant difference between the numbers of YSB (*F*_2,15_ = 0.553, *P* > 0.05) or SSB (*F*_2,15_ = 1.500, *P* > 0.05) egg masses on either IR62, IR64, or *O. rufipogon* in experiments with three rice lines (Fig. 2C). There was also no effect of rice line on the numbers of larvae emerging from YSB egg masses (*F*_2,15_ = 0.288, *P* > 0.05); however, fewer larvae emerged from SSB egg masses on *O. rufipogon* (*F*_2,15_ = 3.750, *P* < 0.05; Fig. 2D).

### Stemborer Performance

**Fig. 3.**
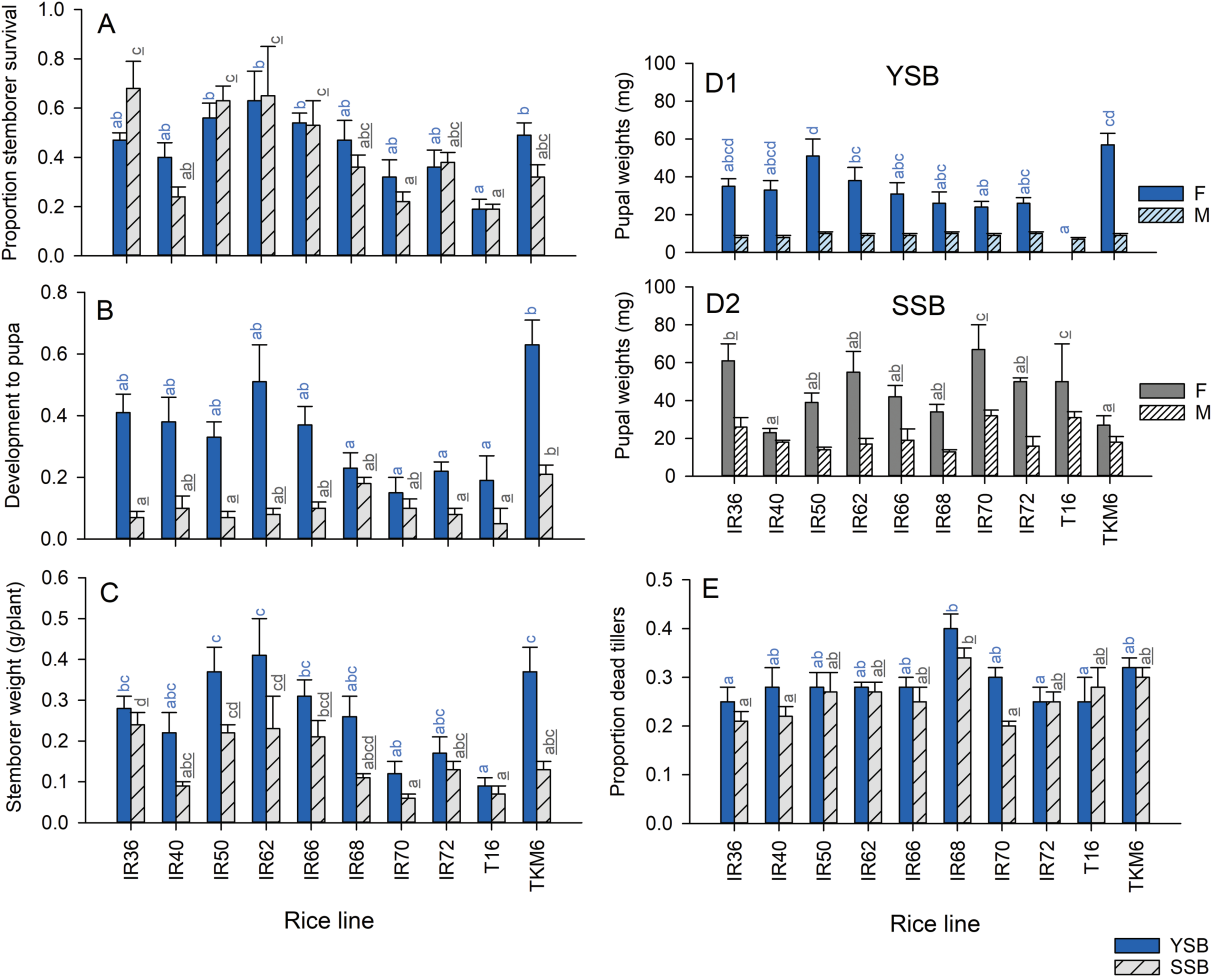
Performance by YSB (blue bars) and SSB (gray bars) and resulting tiller damage to rice seedlings in a screenhouse experiment. Stemborer performance is indicated by (A) the proportions of larvae surviving, (B) the proportion at the pupal stage and (C) total body mass at the end of the experiment. The weights of (D1) YSB pupae and (D2) SSB pupae, and (E) damage to tillers are also indicated. Plants were the same as those used in the oviposition experiments (egg masses were removed before the plants were infested with larvae: see Fig. 2A). Standard errors are indicated, lowercase letters indicate homogenous groups after Tukey’s HSD tests (blue = YSB, gray, underlined = SSB: *P* > 0.05; *n* = 6 for species × variety groups). See online version for colors.

Survival of the larvae of both moth species was affected by host variety, and was relatively low on T16 and IR70 and high on IR50, IR66, and IR72 (SSB: *F*_9,50_ = 7.006, *P* < 0.001, YSB: *F*_9,50_ = 3.575, *P* < 0.01, Fig. 3A). Development was more rapid for both species on TKM6 and slowest on T16 and IR72 (SSB: *F*_9,50_ = 3.137, *P* < 0.01, YSB: *F*_9,50_ = 4.281, *P* < 0.01, Fig. 3B). YSB biomass was greater on TKM6, IR50, and IR62 than on T16, whereas SSB gained greatest biomass on IR36 plants and low biomass on T16 and IR70 (YSB: *F*_1,50_ = 3.575, *P* < 0.01; SSB: *F*_1,50_ = 7.006, *P* < 0.001, Fig. 3C). Females of both species were considerably larger than males (YSB: *F*_1,77_ = 161.216, *P* < 0.001; SSB, *F*_1,68_ = 87.654, *P* < 0.001, Fig. 3D1, D2). YSB pupae were heaviest on IR50 and TKM6 and lightest on T16, but SSB pupae were heaviest on IR70 and T16 (YSB: *F*_9,77_ = 2.968, *P* < 0.01; SSB: *F*_9,68_ = 6.003; *P* < 0.001, Fig. 3D1, D2). The proportion of damaged tillers was highest on IR68 for both species (SSB: *F*_9,50_ = 3.084, *P* < 0.01, YSB: *F*_9,50_ = 2.574, *P* < 0.05, Fig. 3E). Overall, both species performed poorly on T16 (except when considering SSB pupal weights), with both species performing relatively well on IR50, IR62, IR66, and TKM6. SSB also performed relatively well on IR36.

YSB survival was correlated with the final weight of stemborers and the proportion of tillers that were killed in the experiment with 10 varieties (Supp Table S2 [online only]). YSB survival on different varieties was also positively correlated with the numbers of tillers per variety in the microplots (Supp Table S2 [online only]). SSB survival and final stemborer weight were correlated and SSB development time was correlated with the proportion of tillers that had died (Supp Table S3 [online only]); however, there was no relation between tiller number or plant biomass and survival in SSB (Supp Table S3 [online only]).

**Fig. 4.**
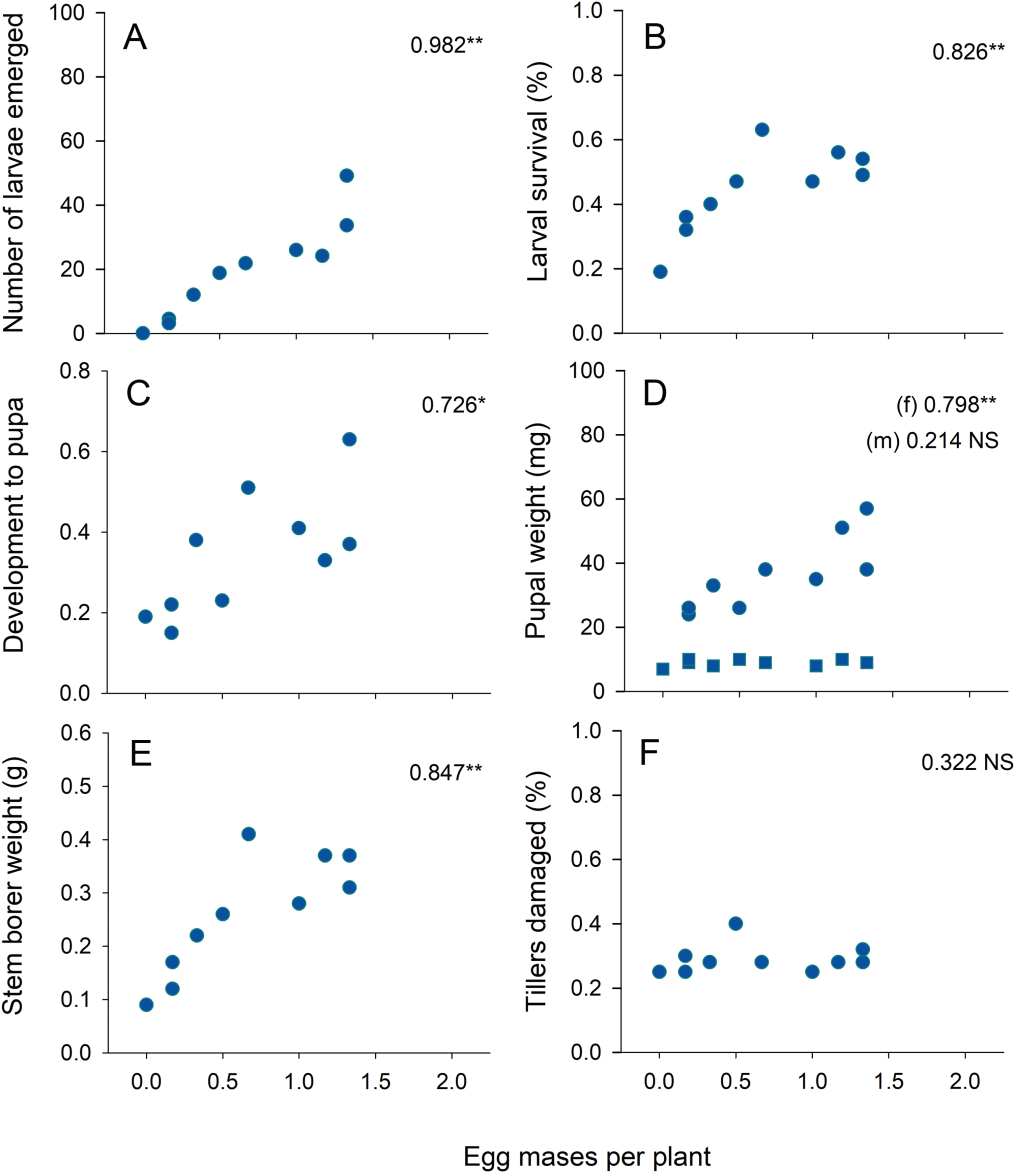
Correlations between the number of YSB egg masses per plant across 10 rice varieties in a choice oviposition experiment (see Fig. 2A) and YSB performance on the same plants (see Figs. 2B and 3). Performance is indicated by (A) neonate emergence, (B) survival to adult, (C) development time as represented by the proportion as pupae at sampling, (D) pupal weight (m = male [squares], f = female [circles]), (E) total stemborer weight per plant, and (F) the proportion of tillers that were damaged. Spearman correlation coefficients are indicated (df = 10, NS = *P* > 0.05, * = *P* < 0.05, ** = *P* < 0.01, *** = *P* < 0.001). There were no significant correlations between SSB oviposition preferences and SSB performance across the rice varieties (Supp Table S1 [online only]).

Oviposition preferences by SSB were not correlated with larval performance (i.e., survival, development rates, pupal weights) on the varieties (Supp Table S1 [online only]). In contrast, neonate numbers, survival, development rates, female pupal weight, and the total biomass of surviving YSB were highly correlated with both the number and proportion of egg masses deposited on the rice plants (Fig. 4A–D; Supp Table S1 [online only]). Male pupal weights and tiller damage were not correlated with oviposition in YSB (Fig. 4F, Supp Table S1 [online only]).

**Fig. 5.**
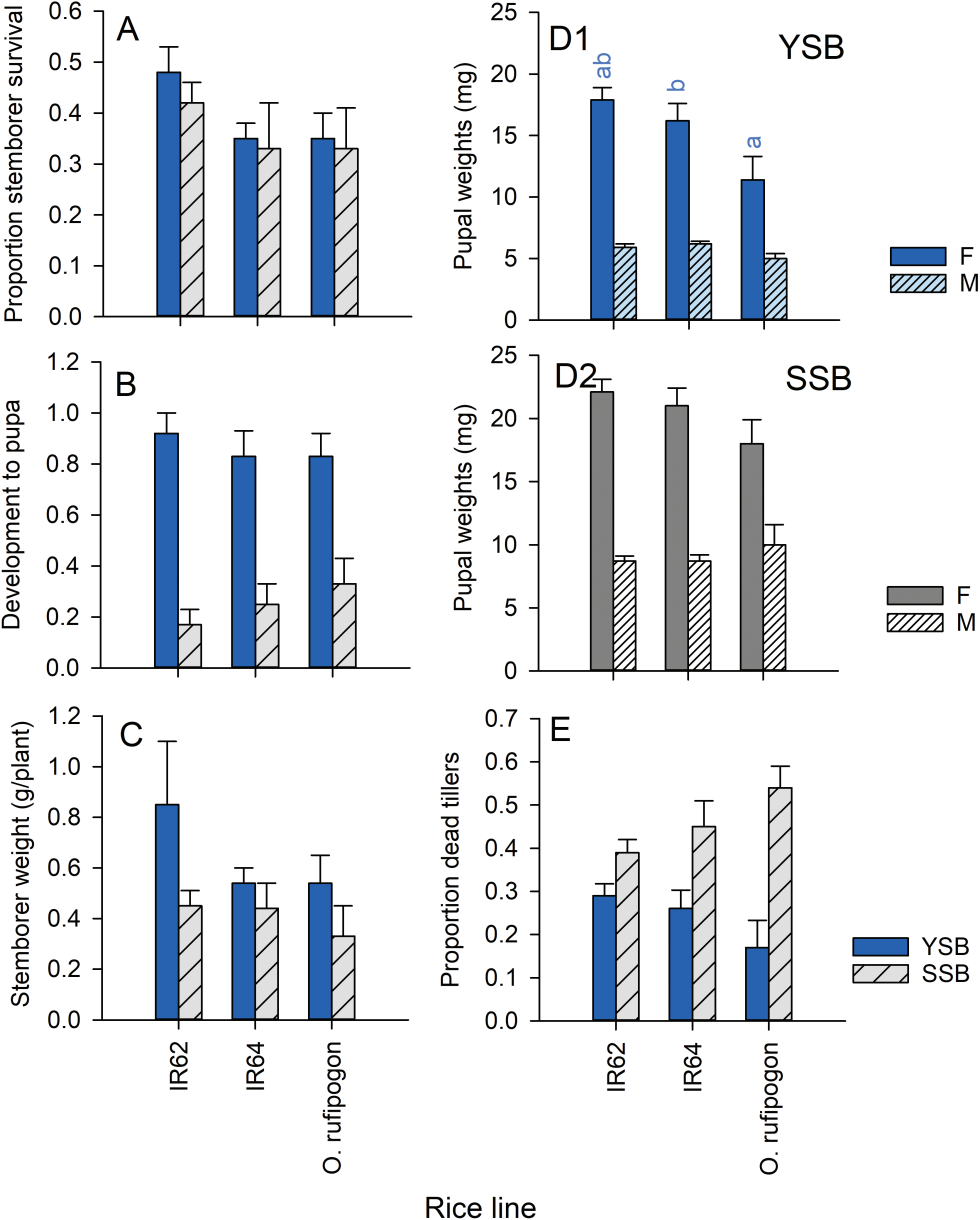
Performance by YSB (blue bars) and SSB (gray bars) and resulting tiller damage to rice seedlings in a screenhouse experiment. Stemborer performance is indicated by (A) the proportions of larvae surviving, (B) the proportion at the pupal stage and (C) total body mass at the end of the experiment. The weights of (D1) YSB pupae and (D2) SSB pupae and (E) damage to tillers are also indicated. Plants were the same as those used in the oviposition experiment (egg masses were removed before the plants were infested with larvae: see Fig. 2C). Standard errors are indicated, lowercase letters indicate homogenous groups after Tukey’s HSD tests (blue = YSB: *P* > 0.05; *n* = 6 for species × variety groups). See online version for colors.

Performance of YSB larvae on *O. rufipogon* was lower than on IR62 and IR64, but differences were generally not statistically significant (*F*_2,15_ survival = 0.439, *F*_2,15_ development time = 0.460, *F*_2,15_ total weight = 0.508) (Fig. 5A–C). Only the weights of male and female pupae were statistically smaller on *O. rufipogon* (males *F*_2,15_ = 4.862, *P* = 0.025, females *F*_2,15_ = 4.330, *P* = 0.033; Fig. 5D1). There was no effect of rice line on the proportions of tillers that were dead at the end of the experiments with YSB (*F*_2,15_ = 2.346, *P* > 0.05; Fig. 5E).

There were no significant reductions in SSB performance on *O. rufipogon* compared to IR62 or IR64 (*F*_2,15_ survival = 0.3.443, *F*_2,15_ development time = 0.266, *F*_2,15_ male pupal weight = 1.253; *F*_2,15_ female pupal weight = 2.331, *F*_2,15_ total weight = 2.331; Fig. 5A–D). There was also no effect of rice line of the proportion of tillers that were damaged (*F*_2,15_ = 1.897, *P* > 0.05; Fig. 5E).

### Correlations Between the Oviposition and Performance of Two Stemborers

**Fig. 6.**
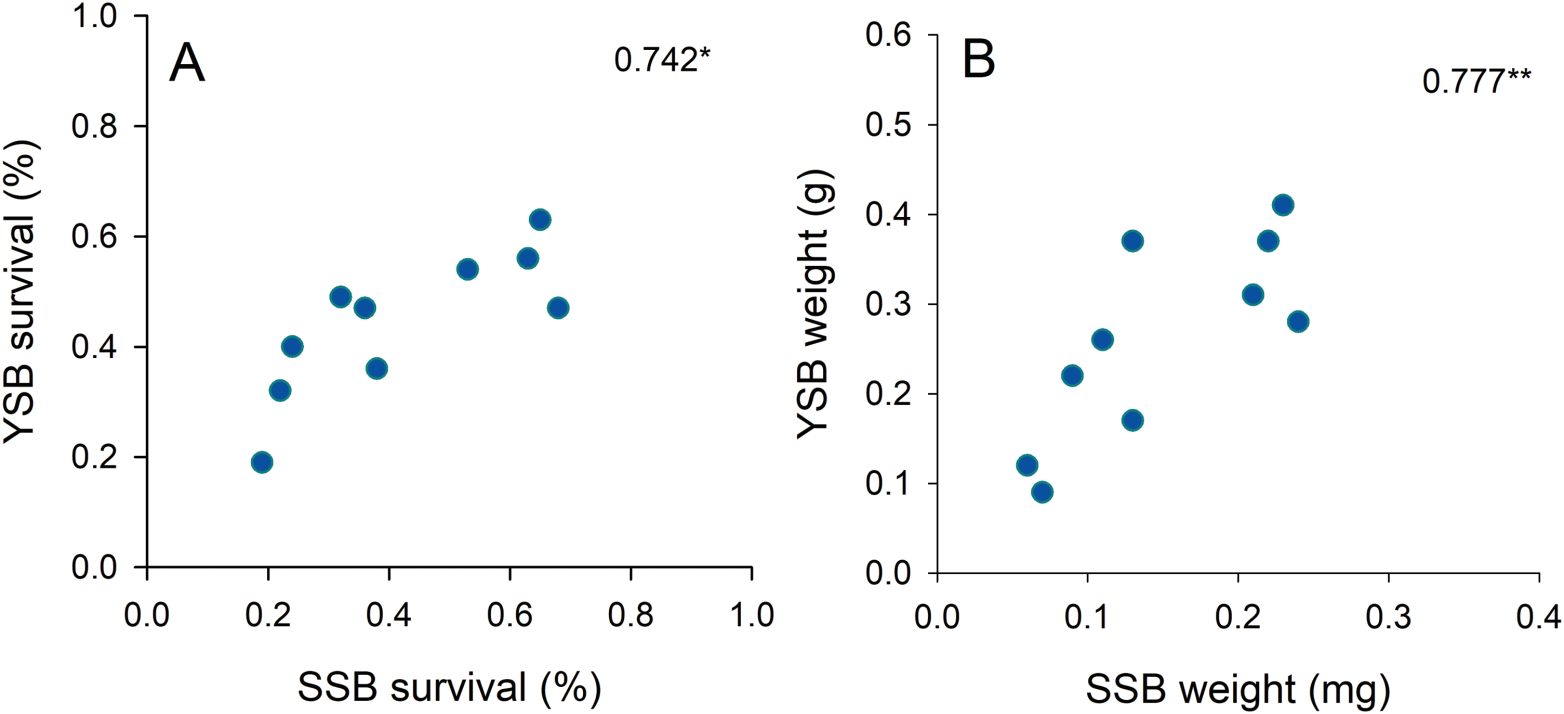
Correlations between (A) the survival of YSB and SSB across 10 rice varieties and (B) the final weight of emerging adults on the varieties. Numbers are Spearman correlation coefficients (df = 10; * = *P* < 0.05, ** = *P* < 0.01).

There were no significant correlations between egg laying by YSB and SSB or the number of larvae of each species emerging from egg masses collected on the 10 rice varieties (egg masses: *Rs*_10_ = −0.103, *P* = 0.777; emerging larvae: *Rs*_10_ = 0.494, *P* = 0.147). Survival (*Rs*_10_ = 0.742, *P* = 0.014, Fig. 6A) and the final biomass (*Rs*_10_ = 0.777, *P* = 0.008, Fig. 6B) of SSB and YSB adults recovered from the plants were correlated. The development times (*Rs*_10_ = 0.294, *P* = 0.410) and female pupal weights (*Rs*_10_ = −0.535, *P* = 0.111) of YSB and SSB across varieties, and the damage caused by each species to the rice varieties (*Rs*_10_ = 0.360, *P* = 0.307) were not correlated.

### Field Experiment With 10 Varieties

Plants were damaged by a range of pests and diseases in the field (Supp Table S4 [online only]), but stemborers were the main source of damage, except in plots with IR50 that were heavily attacked by bacterial blight (*Xanthomonas oryzae*) (Supp Table S4 [online only]). The development of YSB larvae at the field site suggests that YSB achieved two generations in the plots (Supp Fig. S1 [online only]). Only two (wet season) and three (dry season) SSB larvae were found during early season sampling, with numbers building up before subsequent sampling.

**Fig. 7.**
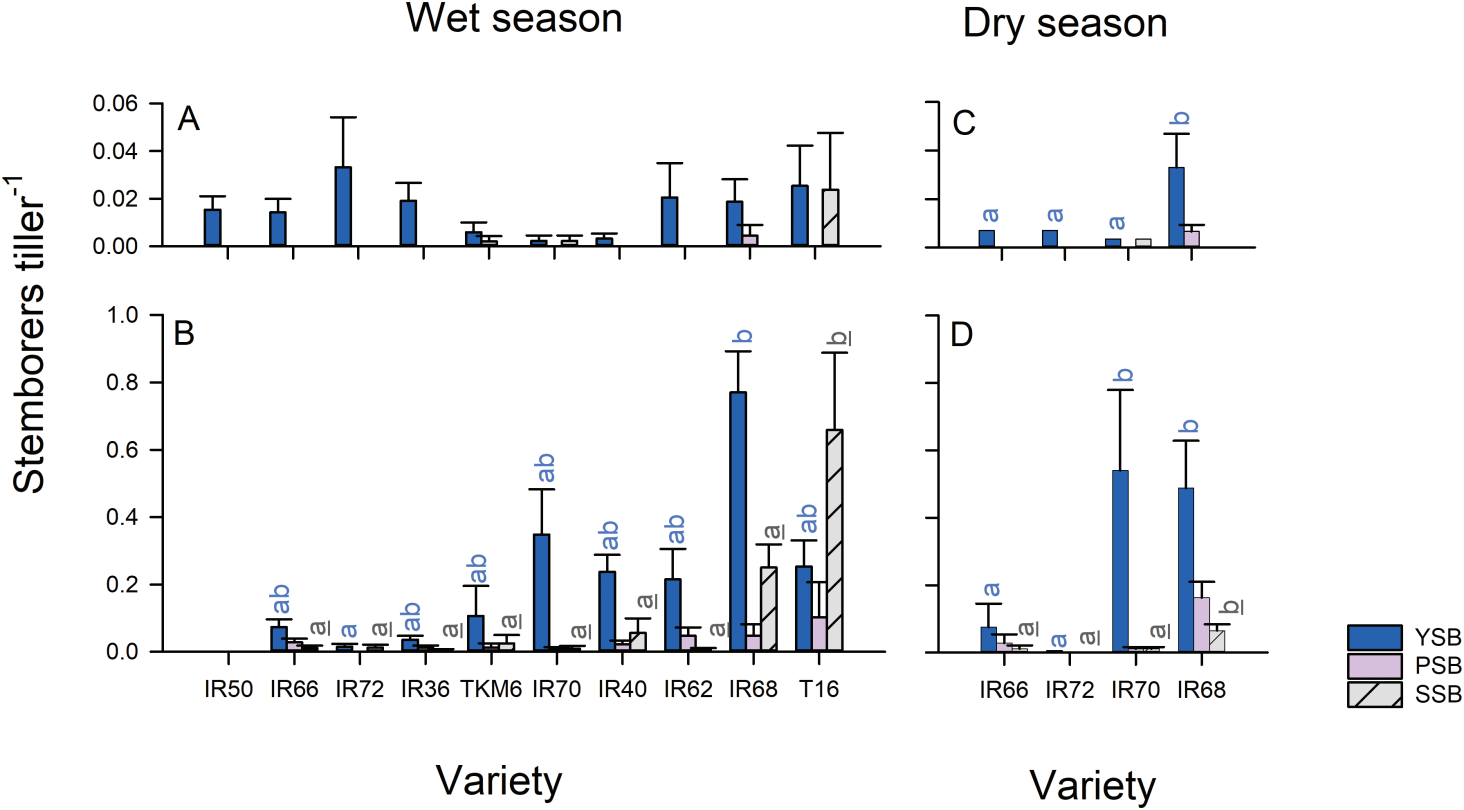
Results from wet season (A, B) and dry season (C, D) field experiments. Data indicate (A) the numbers of stemborers per tiller at (A) 30 DAT and (B) 60 DAT during the wet season, and at (C) 30 DAT and (D) 60 DAT during the dry season. Standard errors are indicated (*n* = 5). Homogenous variety groups are indicated based on Tukey’s HSD tests (*P* < 0.05, blue letters = YSB, gray, underlined letters = SSB). Note that IR50 was excluded from analysis at 60 DAT because of a high incidence of bacterial blight. See online version for colors.

There were no differences in the numbers of egg masses recorded from seedlings of each of the 10 varieties during the wet season experiment (average = 0.04 plant^-1^: *F*_9,50_ = 1.295, *P* > 0.05). There was no significant effect of rice variety on the numbers of larval YSB (*F*_9,50_ = 1.151), PSB (*F*_9,50_ = 0.889) or SSB (*F*_9,50_ = 0.933) occurring in tillers during early wet season sampling (Fig. 7A). At 60 DAT, significantly more YSB larvae occurred on IR68 than IR72 (*F*_8,45_ = 2.488, *P* = 0.025). Significantly more SSB larvae occurred on T16 than on any of the remaining varieties, except IR68 (*F*_8,45_ = 4.450, *P* = 0.001), but there was no effect of variety on the numbers of PSB larvae occurring on the plants (*F*_8,45_ = 1.339, *P* = 0.249; Fig. 7A).

The number of tillers (*P* = 0.010), average tiller weight (*P* = 0.032), and the number of leaves per tiller (*P* = 0.003) were the best predictors of YSB numbers during early sampling (*F*_3,6_ = 12.055, *P* = 0.006). Shoot weight (*P* = 0.009) and leaf weight (*P* = 0.031) were the best predictors of YSB numbers during the later sampling (*F*_2,6_ = 29.304, *P* = 0.001). There were no statistically significant predictors of SSB numbers across varieties during the same sampling.

### Field Experiment With Four Varieties

There were no differences between the numbers of egg masses occurring on seedlings of the four rice varieties during the dry season experiment (average = 0.03 plant^-1^: *F*_3,16_ = 0.326, *P* > 0.05). More YSB larvae (*F*_3,16_ = 5.604, *P* = 0.008) and PSB larvae (*F*_3,16_ = 5.804, *P* = 0.007) occurred on IR68 than on the other three varieties during early sampling. SSB larvae were only detected on IR70 at that time (Fig. 7C). At 60 DAT, more YSB (*F*_3,16_ = 3.704, *P* = 0.034), and SSB (*F*_3,16_ = 7.871, *P* = 0.002) occurred on IR68 compared to the other varieties, and more PSB occurred on IR68 compared to IR72 (*F*_3,16_ = 6.987, *P* = 0.003; Fig. 7D).

Field densities of YSB and SSB eggs and larvae from the wet and dry season experiments were not correlated with screenhouse preferences or any performance parameters from the screenhouse study (−0.678 > *R* < 0.800, all *P*s > 0.05). In particular, the high numbers of SSB on IR68 and T16 in the wet season field experiment contrasted with relatively low preferences for these varieties by the same species in the screenhouse study. There was also no significant correlation between egg laying by moths and larval numbers on varieties in the wet season experiment.

## Discussion

In our screenhouse experiments, we found relatively strong preference–performance coupling in YSB. Several previous studies failed to observe preference–performance coupling for this species (Chandramohan 1983, Riaz et al. 1993; Horgan et al. 2020), although significant correlations between oviposition and damage to rice have been noted in field trials (Kumar 1995, Horgan et al. 2020). Discrepancies between our results and those from previous studies may relate to plant growth conditions that differed between ovipositing adults and developing larvae in previous studies. In our microplots, adult YSB preferentially deposited their egg masses on plants with higher numbers of tillers. Larval survival and development were greatest on these same plants. In contrast to YSB, although SSB performed better on certain rice varieties, and these were often the same varieties on which YSB performed best, SSB adults did not demonstrate clear preferences for any of the varieties in our microplots. This may be related to the nature of the rice varieties we used in our experiments, and does not discount potential preferences by SSB when offered a range of other rice genotypes. For example, Shim (1965) found SSB to display marked preference–performance coupling in a study with rice varieties that included *japonica* and *indica* subspecies. Zhu et al. (2002) also found SSB to oviposit more on ‘new plant type’ (NTP) rice than on semi-dwarf *indica* varieties in field trials in the Philippines. We have also noted SSB to demonstrate significant preferences from among inbred and hybrid rice in choice experiments (unpublished data) and in the present study, fewer SSB larvae emerged from eggs laid on *O. rufipogon* than on two *O. sativa* ssp *indica* varieties from a choice experiment. Although performance was similar on these rice lines in our experiments, previous experiments have indicated SSB to perform poorly on *O. rufipogon* as the wild rice species matures (Reuolin et al. 2019, Horgan et al. 2020). Taken together, these results suggest that YSB preferences are more fine-tuned than those of SSB.

### Potential Traits Influencing Preference–Performance Coupling

Based on a meta-analysis of published studies, Gripenberg et al. (2010) proposed that preference–performance coupling is modified by ecological and/or life-history factors. These authors predicted that coupling would be tighter for species with narrower diets, for species whose offspring have a low dispersal capacity, and for species with gregarious larvae. Only one of these predictions fits our observations of weaker coupling in SSB compared to YSB. Although small numbers of SSB and YSB can survive for extended periods when confined on wild grasses and other non-rice hosts (Demayo et al. 1994, Cohen and Cuong 2002, Cuong et al. 2016), both species are largely oligophagous on rice species (including domesticated and wild rice species (Demayo et al. 1994, Cuong et al. 2016). However, in China, SSB will also oviposit and feed on water-oat (*Zizania latifolia*) (Jiang et al. 2015). This suggests that as a species SSB has a broader diet than YSB and can be predicted to have weaker preference–performance relations among relatively similar rice plant types, as was indicated from our results.

The larvae of SSB, although not gregarious, often occur in larger numbers on individual rice tillers compared to YSB. In our wet and dry season field experiments, 98.8% of YSB-infested tillers, 98.4% of PSB-infested tillers, and 94.0% of SSB-infested tillers contained only a single larva. Larvae of all three stemborer species were present together in a small number of plants—but no tiller contained more than a single species. For SSB, a single tiller could contain up to five larvae. Little is known of the relative dispersal abilities of YSB or SSB, however, Cohen et al. (2000) suggest that neonates of YSB will disperse more than SSB. Observed gregariousness and dispersal might be expected to change under the influence of interspecific competition; however, the results from our single-species experiments suggest that gregariousness and dispersal ability are not useful predictors of relative preference–performance coupling in YSB and SSB.

### Proximate Factors Affecting Between-Plot Distribution

The distribution of larvae across varieties in our field plots suggests that the occurrence of YSB and SSB on different plants was influenced by the nature of the hosts as the crop matured. For example, several varieties in both the wet and dry season field experiments had relatively low densities of both YSB and SSB; these varieties included IR66, IR72, IR36, and TKM6. It is noteworthy that many of these varieties appeared relatively susceptible in the screenhouse experiments. Some of the differences between screenhouse and field results may be related to the relatively advanced development of plants in the field plots at the time of sampling (60 DAT—often at the booting stage) compared to the screenhouse (50 DAT—maximum tillering). This was possibly accelerated by the relatively higher densities of plants in the field trials and the greater intensity of light under field conditions. Meanwhile, IR68, IR70, and T16 had relatively high stemborer densities in the field experiments compared to the other varieties. This again contrasted with the screenhouse results, which indicated IR70 and T16 as among the least suitable hosts for both of the stemborer species. Multiple regression analysis using field data suggested that shoot and tiller weight are among the best predictors of YSB densities: both IR68 and T16 produce heavy tillers and were among the tallest plants from among the varieties exposed in the wet season field experiments (Supp Table S5 [online only]). However, IR68 produces relatively large numbers of tillers during the vegetative growth stage, with reabsorption at later stages (i.e., IR68 tiller number at vegetative stage = 33 plant^-1^, reproductive stage = 17–20 plant^-1^), whereas T16 produces a small number of tillers that gradually gain weight without significant loss of tillers after maximum tillering (i.e., T16 tiller number at vegetative stage = 20 plant^-1^, reproductive stage = 18–19 plant^-1^;Supp Table S5 [online only]).

The numbers of tillers during the vegetative stage were correlated with oviposition by YSB in our screenhouse study and were one of the significant predictors of YSB densities during the early sampling conducted in the wet season field experiment. Meanwhile, tiller number did not correlate with SSB preferences or densities in either the screenhouse or field studies. During our 60 DAT sampling, the average dry weight of individual tillers was greater than 3 g for IR68 and T16, about 2.5 g for IR70 and IR72, and less than 2 g for all other varieties (Supp Table S5 [online only]). The relative distributions of YSB and SSB larvae in our field plots suggest that differences in tillering strategies largely determine the suitability of the plants for either YSB or SSB, with large tillers favoring both species, but with YSB avoiding plants that have low numbers of tillers at early growth stages. This same trend was apparent from experiments conducted by Zhu et al. (2002). In their field study, SSB was more abundant on the NPT IR66011 that had low tillering and larger stems than on the *indica* varieties used in the same experiments. YSB larval densities were correspondingly low on the same NPT variety. Together, the results from Zhu et al. (2002) and from this study suggest that host suitability for developing larvae is the proximate factor determining larval occurrence across varieties in field plots. We did not find evidence of significant preferences by ovipositing YSB or SSB for the most suitable varieties during our field sampling; however, we conducted sampling for eggs during only a single day for both the wet and dry season crops. This was done to better relate our screenhouse and field experiments, but was insufficient to detect significant responses to crop varieties in the field.

### Ultimate Factors Affecting Between-Plot Distribution

We predicted that the species displaying stronger preferences from our screenhouse study would also occur on the most suitable host varieties in the field, with the second species occupying relatively competitor free-plants. Our results indicate that this occurred during our wet season field experiment. Although the most preferred and most suitable plants for YSB from the screenhouse experiments did not correspond with those plants on which YSB larvae were most abundant in the field, the species did, nevertheless, occur at high densities on relatively suitable plants that also had a relatively long field duration (i.e., IR68 and IR70). Horgan et al. (2020) have indicated that crop duration (i.e., crop vulnerability) can decouple the results of stemborer life-history experiments conducted under controlled and field conditions. SSB occurred predominantly on T16 in our wet season experiment. But, SSB egg-laying (numbers of egg masses, but not numbers of emerging larvae), larval survival, and final stemborer biomass were consistently low on T16 in the screenhouse experiments, and the variety has a relatively short field duration. This suggests that in our wet season field experiment, SSB occurred predominantly on a relatively unsuitable host (compared to IR68 and IR70). Our screenhouse experiments indicated that the variety T16 was avoided by ovipositing YSB due to low tillering at early plant stages. Such avoidance by YSB of plants with low numbers of tillers during early stages of the wet season experiment would have created a space among the T16 field plots for SSB that was relatively free of damage and allowed SSB to become the dominant species in those plots. This idea is further supported by evidence from field experiments conducted by Zhu et al. (2002) who found high numbers of SSB in NPT rice lines that were relatively free of YSB.

There are several examples of herbivores avoiding plants previously attacked by potential competitors. Evidence suggests that changes in plant volatiles emitted from damaged plants can alter the behavior of subsequent herbivore colonizers (Dicke 2000, Dicke and van Loon 2000, Fatouros et al. 2012). Additional research into the co-occurrence of YSB and SSB as related to the timing of egg laying, the semiochemical cues involved in host finding, and post-emergence dispersal behaviors is required to further elucidate potential ultimate factors affecting the relative distributions of each species at plant and plot scales. However, it is unlikely that the availability of competitor-free plants would affect the relative distributions of these moths at larger field scales (i.e., hectares of rice). At such large scales, the predominant varieties of rice, or the prevalence of agricultural practices that affect rice tillering or rice stem thickness, probably alter the distributions of these two stemborers by favoring one species over the other. Field-based studies of stemborer preference–performance at landscape scales are required to better understand such area-wide management effects.

### Recommendations

Rice breeders, interested in developing rice varieties with resistance to stemborers have generally focused on single species from target stemborer assemblages (74% of 207 published papers: Horgan unpublished). SSB has been the predominant species studied in rice breeding programs in recent years. Results from comparative studies of SSB and YSB, including the present study, suggest that whereas larval performance in both species, or plant responses to attack by both species may be correlated across varieties, preferences are not (Horgan et al. 2020). Therefore, SSB can be a suitable surrogate for YSB in breeding programs, but only during performance experiments (i.e., not for preference studies). Furthermore, our experiments with SSB indicate that the results from screenhouse and field studies may be decoupled due to interspecific stemborer interactions and weak preference–performance coupling, as well as physiological changes to plants grown under different experimental conditions. A comparison of results from our wet season and dry season experiments indicates that such decoupling will also depend on the availability of varieties in the screening trails that are nonpreferred by the dominant species, but highly suitable for a secondary species. Therefore, information on preference–performance coupling should be built in to breeding programs aimed at complexes of stemborers. Finally, evidence of shifts in the abundance of SSB and YSB at regional scales suggests that the relative distributions of these two species are condition dependent, including dependent on environmental conditions such as irrigation practices and climate (Kiritani 1990, Litsinger et al. 2006). Our results indicate a continuing role for conventionally bred stemborer ‘resistant’ varieties in ecologically-based rice pest management (Horgan 2018, Horgan et al. 2019). The careful choice of rice varieties could therefore increase the resilience of rice production systems against undesirable shifts in stemborer assemblages (e.g., avoiding dominance by a more damaging species) that result from changing environmental conditions.

## Supplementary Material

nvab034_suppl_Supplementary_Information
