# Supplementary material for: Differences Between the Strength of Preference–Performance Coupling in Two Rice Stemborers (Lepidoptera: Pyralidae, Crambidae) Promotes Coexistence at Field-Plot Scales
Source: Environ Entomol. 2021 Apr 28;50(4):929–39. doi: 10.1093/ee/nvab034 (PMC8359816; doi:10.1093/ee/nvab034)
Supplement: nvab034_suppl_Supplementary_Information [file nvab034_suppl_supplementary_information.docx]

SUPPLEMENTARY INFORMATION

Table S1. Spearman correlation coefficients based on preference-performance data from the screen house experiment^1^

| Performance | YSB^2^ |  | SSB |  |
| --- | --- | --- | --- | --- |
|  | Number of masses | Proportion of masses | Number of masses | Proportion of masses |
| Larvae emerged | 0.982*** | 0.979*** | 0.494 | 0.275 |
| Survival to adult | 0.826*** | 0.771** | -0.067 | 0.350 |
| Development rate | 0.726** | 0.711** | 0.292 | -0.072 |
| Pupal weight (female) | 0.798** | 0.713** | -0.518 | -0.416 |
| Pupal weight (male) | 0.214 | 0.089 | -0.119 | -0.187 |
| Survivor biomass | 0.847*** | 0.784** | 0.129 | 0.434 |
| Damaged tillers | 0.322 | 0.248 | -0.132 | -0.298 |

1: See data for YSB in Fig. 4

2: ** = *P* <0.01, *** = *P* <0.001 (df = 10)

**Table S2:** Spearman rank correlations between plant characteristics and fitness traits for YSB (see Fig. 3)

| Parameters | YSB number of dead tillers | YSB proportion dead heart | YSB proportion of insects surviving | YSB weight of insects surviving | YSB proportion adults | Plant biomass | Tiller number |
| --- | --- | --- | --- | --- | --- | --- | --- |
| YSB number of dead tillers^1^ | - | 0.653 (0.041)* | 0.462 (0.179) | 0.401 (0.250) | 0.091 (0.803) | 0.164 (0.651) | 0.539 (0.108) |
| YSB proportion dead heart^1^ | - | - | 0.267 (0.456) | 0.283 (0.429) | 0.283 (0.429) | (-0.025 (0.945) | (-0.006 (0.986) |
| YSB proportion of insects surviving^1^ | - | - | - | 0.976 (0.001)*** | 0.699 (0.024)* | 0.444 (0.199) | 0.626 (0.053)* |
| YSB weight of insects surviving^1^ | - | - | - | - | 0.802 (0.005)*** | 0.432 (0.213) | 0.535 (0.111) |
| YSB proportion adults^1^ | - | - | - | - | - | 0.139 (0.701) | 0.224 (0.533) |
| Plant biomass^1^ | - | - | - | - | - | - | 0.152 (0.676) |

*** = P ≤ 0.005; * = P ≤ 0.05; numbers in parentheses are calculated *P*-values

**Table S3:** Spearman rank correlations between plant characteristics and fitness traits for SSB (see Fig. 3)

| Parameters | SSB number of dead tillers | SSB proportion dead heart | SSB number of insects surviving | SSB weight of insects surviving | SSB proportion adults | Tiller number |
| --- | --- | --- | --- | --- | --- | --- |
| SSB number of dead tillers^1^ | - | 0.450 (0.192) | 0.511 (0.132) | 0.269 (0.452) | 0.375 (0.286) | 0.466 (0.174) |
| SSB proportion dead heart^1^ | - | - | 0.200 (0.580) | 0.165 (0.649) | 0.741 (0.014)** | -0.395 (0.258) |
| SSB proportion of insects surviving^1^ | - | - | - | 0.924 (0.001)*** | -0.130 (0.721) | 0.438 (0.206) |
| SSB weight of insects surviving^1^ | - | - | - | - | -0.165 (0.650) | 0.336 (0.342) |
| SSB proportion adults^1^ | - | - | - | - | - | -0.176 (0.626) |

*** = P ≤ 0.005; ** = P ≤ 0.01; numbers in parentheses are calculated *P*-values

**Table S4:** Damage to rice foliage during the field experiment

| Sample | Variety | Leaf damage^1^ |  |  |  |  |
| --- | --- | --- | --- | --- | --- | --- |
|  |  | Whorl maggot | Leaf folder | Caterpillars | Bacterial blight | Leaf spot |
| Early September |  |  |  |  |  |  |
|  | IR36 | 0.62 (0.04) | 0.40 (0.05) | 0.02 (0.01) | 0.03 (0.01) | <0.01 (0.00) |
|  | IR40 | 0.45 (0.03) | 0.22 (0.02) | 0.02 (0.01) | <0.01 (0.00) | <0.01 (0.00) |
|  | IR50 | 0.55 (0.05) | 0.30 (0.04) | 0.03 (0.01) | 0.19 (0.04) | <0.01 (0.00) |
|  | IR62 | 0.65 (0.10) | 0.36 (0.05) | 0.01 (0.00) | 0.01 (0.00) | <0.01 (0.00) |
|  | IR66 | 0.52 (0.04) | 0.27 (0.04) | 0.03 (0.01) | <0.01 (0.00) | 0.04 (0.03) |
|  | IR68 | 0.46 (0.10) | 0.28 (0.04) | 0.01 (0.00) | <0.01 (0.00) | 0.02 (0.01) |
|  | IR70 | 0.52 (0.03) | 0.27 (0.05) | 0.01 (0.01) | 0.01 (0.01) | <0.01 (0.00) |
|  | IR72 | 0.62 (0.03) | 0.20 (0.02) | 0.03 (0.01) | <0.01 (0.00) | <0.01 (0.00) |
|  | T16 | 0.44 (0.06) | 0.27 (0.06) | 0.02 (0.01) | 0.01 (0.00) | <0.01 (0.00) |
|  | TKM6 | 0.43 (0.06) | 0.11 (0.03) | <0.01 (0.00) | 0.06 (0.03) | 0.01 (0.01) |
| Late September |  |  |  |  |  |  |
|  | IR36 |  | 0.28 (0.02) | 0.40 (0.09) | 0.03 (0.01) | 0.00 (0.00) |
|  | IR40 |  | 0.21 (0.06) | 0.16 (0.04) | <0.01 (0.00) | 0.00 (0.00) |
|  | IR50 |  | 0.14 (0.03) | 0.27 (0.09) | 0.15 (0.06) | 0.01 (0.01) |
|  | IR62 |  | 0.44 (0.06) | 0.27 (0.05) | 0.02 (0.00) | 0.00 (0.00) |
|  | IR66 |  | 0.52 (0.16) | 0.29 (0.06) | 0.03 (0.02) | 0.08 (0.04) |
|  | IR68 |  | 0.48 (0.12) | 0.27 (0.04) | 0.03 (0.02) | <0.01 (0.00) |
|  | IR70 |  | 0.27 (0.04) | 0.28 (0.05) | 0.02 (0.01) | 0.00 (0.00) |
|  | IR72 |  | 0.16 (0.03) | 0.26 (0.07) | 0.03 (0.01) | <0.01 (0.00) |
|  | T16 |  | 0.25 (0.03) | 0.24 (0.09) | 0.02 (0.02) | 0.00 (0.00) |
|  | TKM6 |  | 0.07 (0.04) | 0.19 (0.09) | 0.05 (0.01) | 0.00 (0.00) |

1: Standard errors are indicated in parentheses (N = 6)

**Table S5:** Traits of rice plants from field plots with ten rice varieties

| Sample | Variety | Number of tillers/plant^1^ | Plant height (cm)^1^ | Leaves/tiller^1^ | Shoot biomass (g dry weight) ^1^ | Leaf biomass (g dry weight)/tiller^1^ | Panicle biomass (g dry weight) ^1^ |
| --- | --- | --- | --- | --- | --- | --- | --- |
| Early September |  |  |  |  |  |  |  |
|  | IR36 | 51.88 (1.73) | 81.73 (1.92) | 4.24 (0.08) | 17.10 (1.06) | 11.86 (0.46) |  |
|  | IR40 | 51.13 (1.19) | 71.96 (2.51) | 3.92 (0.04) | 13.92 (0.80) | 10.13 (0.38) |  |
|  | IR50 | 48.08 (2.97) | 77.63 (1.34) | 4.04 (0.03) | 17.96 (1.09) | 9.92 (0.82) |  |
|  | IR62 | 40.83 (1.63) | 84.76 (1.40) | 4.41 (0.05) | 17.34 (1.06) | 11.45 (0.53) |  |
|  | IR66 | 43.71 (3.77) | 82.88 (2.05) | 4.29 (0.06) | 18.16 (1.71) | 13.85 (1.36) |  |
|  | IR68 | 32.75 (1.91) | 95.64 (1.94) | 4.45 (0.05) | 17.75 (0.91) | 14.32 (1.05) |  |
|  | IR70 | 44.71 (1.76) | 78.79 (2.25) | 4.13 (0.08) | 16.58 (0.96) | 11.86 (1.20) |  |
|  | IR72 | 32.96 (1.23) | 80.89 (1.18) | 4.60 (0.04) | 22.49 (1.40) | 9.78 (0.22) |  |
|  | T16 | 20.04 (1.43) | 95.12 (1.65) | 4.64 (0.06) | 16.58 (0.71) | 9.50 (0.41) |  |
|  | TKM6 | 46.21 (1.76) | 106.78 (3.17) | 3.98 (0.03) | 18.36 (0.96) | 11.01 (0.19) |  |
| Late September |  |  |  |  |  |  |  |
|  | IR36 | 29.54 (1.25) | 98.78 (1.02) | 4.12 90.07) | 56.02 (2.88) | 3.12 (0.00) | 5.54 (5.54) |
|  | IR40 | 35.71 (1.47) | 114.73 (1.55) | 4.74 (0.04) | 67.33 (3.43) | 10.00 | 0.00 (0.00) |
|  | IR50 | 40.38 (4.96) | 83.77 (0.58) | 3.88 (0.07) | 17.99 (1.35) | 9.08 (0.95) | 13.23 (1.28) |
|  | IR62 | 29.04 (1.43) | 112.78 (1.88) | 4.74 (0.06) | 40.47 (6.61) | 12.13 (0.22) | 19.01 (6.26) |
|  | IR66 | 28.54 (0.97) | 107.57 (1.58) | 4.53 (0.06) | 59.75 (7.92) | 12.81 (1.31) | 30.39 (9.95) |
|  | IR68 | 20.29 (1.10) | 118.92 (3.37) | 5.25 (0.07) | 64.47 (3.21) | 10.00 | 0.00 (0.00) |
|  | IR70 | 27.29 (1.02) | 114.08 (1.02) | 4.91 (0.08) | 70.34 (3.11) | 10.00 | 0.00 (0.00) |
|  | IR72 | 24.79 (1.06) | 95.71 (1.84) | 4.19 (0.09) | 59.50 (3.85) | 8.68 (0.48) | 12.05 (7.64) |
|  | T16 | 18.79 (0.48) | 119.25 (0.61) | 4.25 (0.13) | 57.48 (3.66) | 5.99 (0.00) | 1.87 (1.87) |
|  | TKM6 | 36.42 (2.79) | 129.03 (1.35) | 3.91 (0.06) | 55.22 (2.39) | 10.00 | 4.19 (4.19) |
| 1: Standard errors are indicated in parentheses (N = 6) | | | | | | | |

**Figure S1:** Life stages of YSB during the wet season field experiment (see Fig. 7A, B) indicating at least two generations. L1 = first instar, L2 = second instar, etc., P = pupa. Standard errors are indicated (N = 6).
